# New Advances in Elastomer Materials and Its Composites

**DOI:** 10.3390/ma19061061

**Published:** 2026-03-11

**Authors:** Magdalena Maciejewska

**Affiliations:** Institute of Polymer and Dye Technology, Lodz University of Technology, Stefanowskiego Street 16, 90-537 Lodz, Poland; magdalena.maciejewska@p.lodz.pl

Elastomers are a class of polymers distinguished by their ability to undergo large elastic deformation and return to their original shape once the applied stress is removed. This unique behavior arises from their molecular structure, which consists of long, flexible polymer chains lightly crosslinked to form a three-dimensional network [1,2]. Elastomers and their composites are critically important because they enable systems to function under real-world dynamic conditions. Their combination of elasticity, durability, chemical resistance, and tunability makes them foundational materials across engineering, healthcare, transportation, and advanced technological applications [3,4]. However, the rapid advancement of modern technologies compels manufacturers to develop elastomer composites with increasingly sophisticated and specialized functional properties [5,6]. At the same time, environmental considerations related to production processes and the end-of-life management of rubber products have become equally significant [7,8,9,10]. As a result, elastomer materials have emerged in recent years as some of the most widely utilized and fastest-evolving engineering materials worldwide. This Special Issue (SI), “New Advances in Elastomer Materials and Its Composites”, presents current trends in the development and research of new elastomeric materials and other polymer biomaterials with controlled elasticity, taking into account ecological aspects and bio-waste management. This Editorial summarizes the five research articles included in this SI.

The space industry is one of the applications of polymer and elastomer composites that requires advanced properties. Regarding the rapid advancement of space technology and space-based optical systems, high-resolution Earth observation and remote-sensing constellations have become a dominant trend in modern aerospace development [11]. Carbon-fiber-reinforced polymers (CFPR) possess great potential use in this field. The high specific stiffness, thermal stability and extensive design possibilities of this type of materials make it advantageous over traditional materials such as glass and metal [12]. CFPR could be successfully applied to produce mirrors of high shape accuracy for reflective optical systems used in space cameras. Thus, Fan et al. [13] investigated ways to reduce the fiber print-through (FPT) effect that appeared on the surface of CFRP mirrors during its curing and replication processes. The CFRP mirror replication technique involves heating the resin matrix to bond it to the fibers. The FPT phenomenon refers to the unwanted surface pattern or roughness caused by reinforcement fibers becoming visible through the resin matrix after curing, which negatively affects the optical quality of CFRP mirror surfaces. In this work, the authors first simulated and analyzed the underlying causes of FPT formation. They identified that the manner in which the resin cured against the mold and the pressure distribution during processing significantly influenced the severity of the print-through phenomenon. Based on this understanding, an improved replication technique involving the controlled application of pressure during curing and a specifically designed mold to assist in pressure distribution was proposed. The experimental results demonstrated that the improved process effectively reduced fiber print-through patterns, producing a smoother mirror surface compared with the conventional method. Applying calibrated pressure during curing was demonstrated to be particularly beneficial in lowering surface roughness and enhancing overall surface quality.

Currently, significant emphasis is placed on environmental protection. As a result, the demand for innovative materials capable of replacing those produced from non-renewable, petroleum-based resources continues to grow annually [14]. Therefore, biomaterials, which are materials containing one or more constituent phases originating from natural sources, are also becoming an increasingly popular and interesting subject of research in the field of elastomeric materials [15]. The use of plant-derived bioadditives as fillers (i.e., biofillers) is one of the trends in elastomeric biocomposites [16,17,18]. The study by Malicka et al. [19] focused on the development of eco-friendly elastomeric materials by incorporating biofillers derived from coffee and tea waste into a thermoplastic elastomer based on an ethylene–norbornene (EN) copolymer. The main goal was to investigate how these plant-based waste fillers, combined with conventional fillers (montmorillonite, silica, and cellulose), affected the properties and stability of the polymer composites, particularly after exposure to ultraviolet (UV) aging cycles. The evaluation of the anti-aging influence of coffee and tea waste when used simultaneously as stabilizers and fillers was a key novel aspect of the research. Results showed that combining coffee waste with silica, montmorillonite and cellulose limited the migration of fatty acids and other compounds from the biofillers to the polymer surface. Additionally, all coffee- and tea-based biofillers were effective in stabilizing the polymer against degradation under prolonged UV exposure. The findings highlight the significance of plant-waste biofillers in affecting both the properties and degradability of synthetic elastomeric materials, pointing to their potential use in more sustainable material systems.

As far as biomaterials are concerned, a material that attracts increasing interest from researchers and offers opportunities for a wide range of potential applications is nanocellulose, particularly cellulose nanocrystals (CNCs) and nanofibrillated cellulose (CNFs), which have been widely studied as reinforcing fillers in elastomer composites offering potential performance gains with a smaller ecological footprint compared with conventional fillers [20]. In addition to plant-derived cellulose, bacterial nanocellulose (BNC) is becoming increasingly popular [21]. Compared with plant cellulose, BNC provides a higher purity, better mechanical performance, improved barrier properties, high water retention and excellent transparency, making it a promising material for sustainable food packaging and biomedical applications. The study by Lisowski et al. [22] presents the development of a novel, eco-friendly flexible leather-like material derived from BNC, with the aim of replacing both traditional animal leather and synthetic polymer leather products. A key challenge with BNC is that it becomes stiff and brittle when dried, which limits its practical use in flexible applications. To overcome this, the researchers explored bio-based plasticization strategies using vegetable oils, including rapeseed, linseed, and grapeseed oils, that could improve the flexibility and mechanical performance of BNC without relying on synthetic additives. Rapeseed oil was most effective, yielding a biomaterial with improved strength and elasticity that approached the qualities needed for leather-like applications.

The use of natural fillers of mineral origin is another developing trend in environmentally friendly elastomer composites [23,24]. One such filler is hydroxyapatite (HAP), which is a naturally occurring mineral composed of calcium hydroxyphosphate. The fundamental challenge in obtaining elastomer composites with satisfactory functional properties is ensuring homogeneous dispersion of this filler in the elastomer matrix [25,26]. The study by Maciejewska et al. [27] aimed to develop and characterize acrylonitrile–butadiene rubber (NBR) composites filled with HAP to improve their vulcanization behavior, thermal stability, and flammability. Several additives were introduced—including a silane coupling agent ((3-aminopropyl)-triethoxysilane), an ionic liquid (1-decyl-3-methylimidazolium bromide), and a surfactant (cetyltrimethylammonium bromide)—to enhance the dispersion of the HAP particles within the elastomer matrix. The research demonstrated that the dispersing agents helped to improve the compatibility between the filler and the rubber matrix, resulting in a better filler distribution and modified vulcanization characteristics. The presence of HAP significantly influenced the curing behavior—increasing optimum vulcanization time—but the use of the ionic liquid and surfactant reduced both the vulcanization temperature and time needed. Importantly, the inclusion of HAP significantly enhanced thermo-oxidative aging resistance and reduced the flammability of the NBR composites compared with unfilled NBR, without adversely affecting their chemical resistance or damping properties. These results suggest that HAP can serve as an effective mineral filler for elastomeric materials to improve fire safety and durability.

Fire safety is critical for building materials, electronics, transportation, textiles, or packaging in which polymers, including elastomers, are widely used. Most polymers are combustible by nature since they are primarily organic compounds composed of carbon and hydrogen. This makes them inherently flammable [28,29]. Controlling their flammability via additives, mineral fillers, or chemical modification is a standard practice in materials engineering. This is especially important for polyurethanes (PUR), which are characterized by their low flame resistance and widely used as building materials [30,31,32]. In terms of fire hazards, not only is the flammability of polyurethane materials important but the emission of smoke and gases during combustion as well, in addition to the toxic combustion by-products. Therefore, Głowacki et al. [33] explored the effects of phosphine compounds, which are also in synergistic systems with organophosphorus compounds, on the properties of PUR composites, with particular emphasis on their fire hazard and biocompatibility. The results indicated that introducing these phosphorus-based flame retardants could enhance the thermal resistance of the flexible PUR foams and reduce fundamental flammability, suggesting that the improved fire performance of the material compared with unmodified foams. However, the influence on smoke production and gaseous emissions was not uniformly positive. In some cases, while concentrations of certain harmful combustion products like CO and HCN were lowered, the formulations simultaneously led to higher amounts of carbon dioxide (CO_2_) and nitrogen oxides (NO_x_) as well as larger smoke densities during burning, highlighting a trade-off between fire resistance and smoke/toxic gas generation. This highlights the importance of a balanced approach when developing safer polymeric materials that must meet both fire safety and environmental emission criteria.

To summarize, elastomer composites are expected to experience dynamic growth over the next few years, driven primarily by the increasing demand for lightweight, durable, flame resistant and high-performance materials. At the same time, advances in nanotechnology are enabling the development of elastomer composites reinforced with nanoscale fillers, which significantly improve functional properties such as conductivity, barrier performance, and durability. Sustainability is of equal importance. Growing regulatory pressure and environmental awareness are fostering the development of eco-friendly elastomer biocomposites, bio-based elastomers, recyclable thermoplastic elastomer composites, and improved rubber recycling technologies. Therefore, sustainability, multifunctionality, and safety are key trends driving development in the field of advanced elastomer composites.
